# Supplementary material for: Potent neutralization of Marburg virus by a vaccine-elicited monoclonal antibody
Source: bioRxiv. 2025 May 18:2025.05.14.654121. Preprint. [Version 1] doi: 10.1101/2025.05.14.654121 (PMC12132480; doi:10.1101/2025.05.14.654121)
Supplement: 1 [file NIHPP2025.05.14.654121V1-supplement-1.pdf]

bioRxiv preprint doi: https://doi.org/10.1101/2025.05.14.654121; this version posted May 18, 2025. The copyright holder for this preprint (which was not certified by peer review) is the author/funder, who has granted bioRxiv a license to display the preprint in perpetuity. It is made available under aCC-BY 4.0 International license.

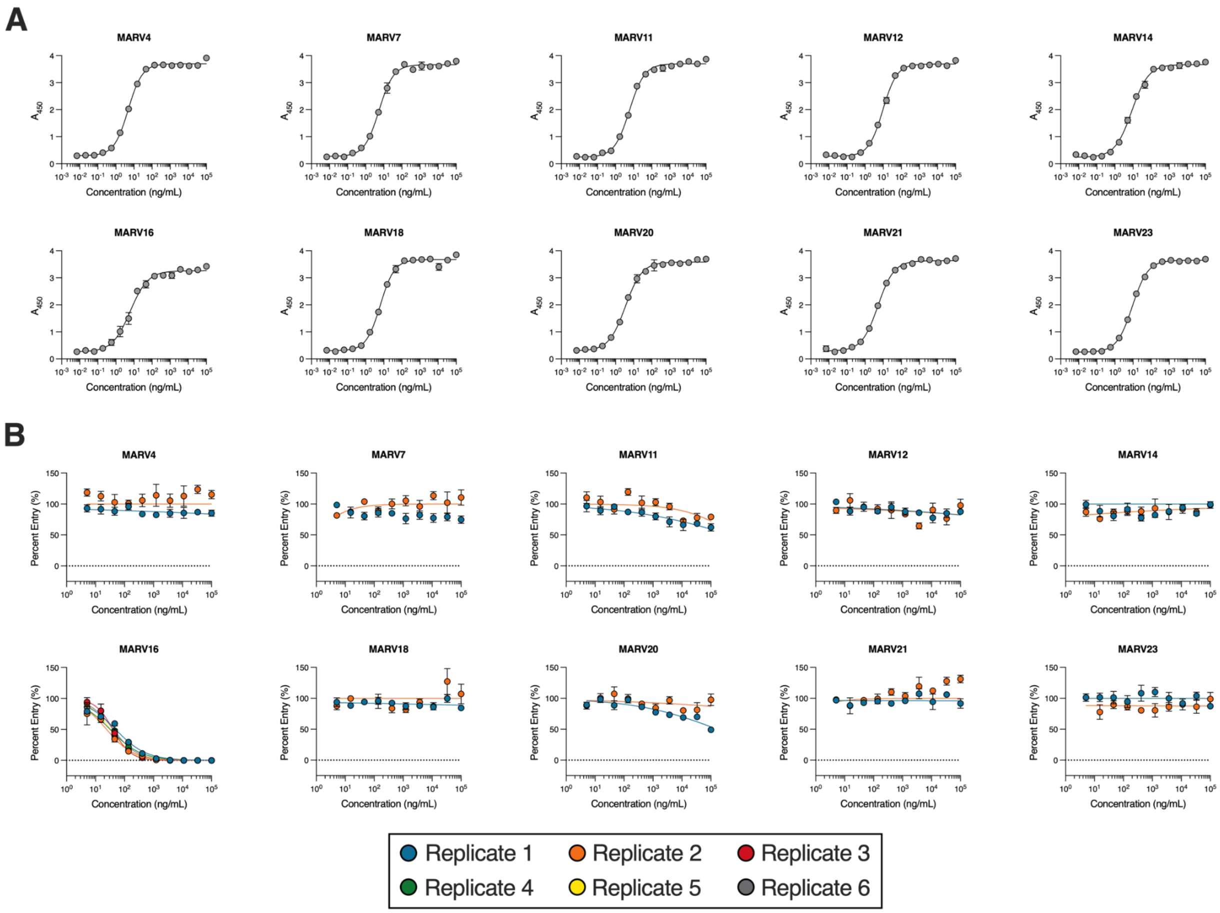


**Figure S1.** Dose-response curves for the ELISAs against the MARV GPΔMuc ectodomain (A) and neutralization assays (B) against VSV pseudotyped with MARV/Musoke GP for the 10 antibodies discovered from the immunization study using the ATX-GK mouse. Two biological replicates were performed for the ELISAs using distinct batches of proteins and antibodies. Two technical replicates were performed per biological replicate. Data presented are from one representative biological replicate and presented as mean ± standard error from the two technical replicates. Two to six biological replicates were performed for the neutralization assays using distinct batches of antibodies and pseudoviruses. Three technical replicates were performed per biological replicate. Data from all biological replicates are shown and presented as mean ± standard error from the three technical replicates.

bioRxiv preprint doi: https://doi.org/10.1101/2025.05.14.654121; this version posted May 18, 2025. The copyright holder for this preprint (which was not certified by peer review) is the author/funder, who has granted bioRxiv a license to display the preprint in perpetuity. It is made available under aCC-BY 4.0 International license.

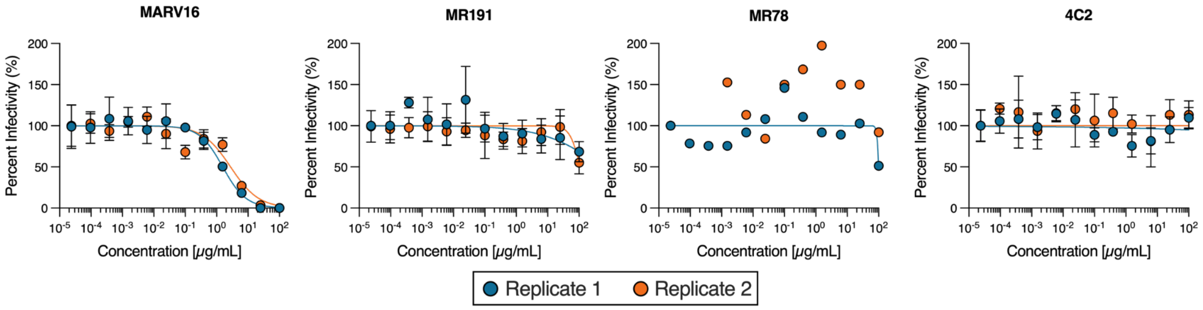


**Figure S2.** Dose-response curves for plaque reduction neutralization tests for MARV16, MR78, MR191, and 4C2, conducted using authentic MARV/Musoke. Two biological replicates were performed with one to three technical replicates using distinct batches of monoclonal antibodies. Data are shown as the mean ± standard error of the technical replicates.

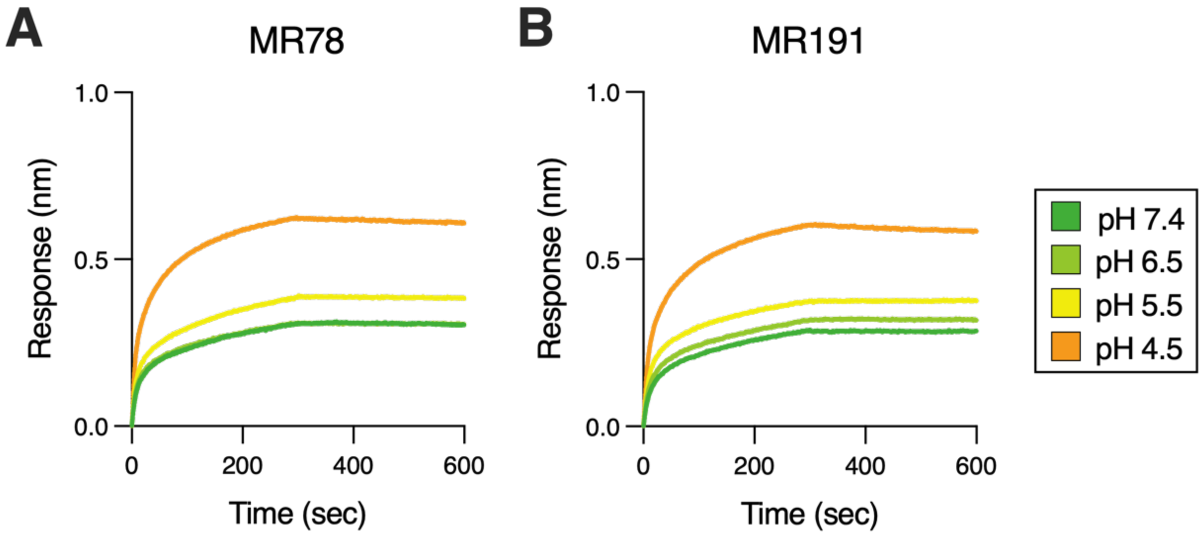


**Figure S3.** Binding of MR78 (A) and MR191 (B) IgGs at a concentration of 100 nM to immobilized MARV GPΔMuc at the indicated pH as measured by biolayer interferometry. Data are representative of two biological replicates using distinct batches of protein and antibodies.

bioRxiv preprint doi: https://doi.org/10.1101/2025.05.14.654121; this version posted May 18, 2025. The copyright holder for this preprint (which was not certified by peer review) is the author/funder, who has granted bioRxiv a license to display the preprint in perpetuity. It is made available under aCC-BY 4.0 International license.

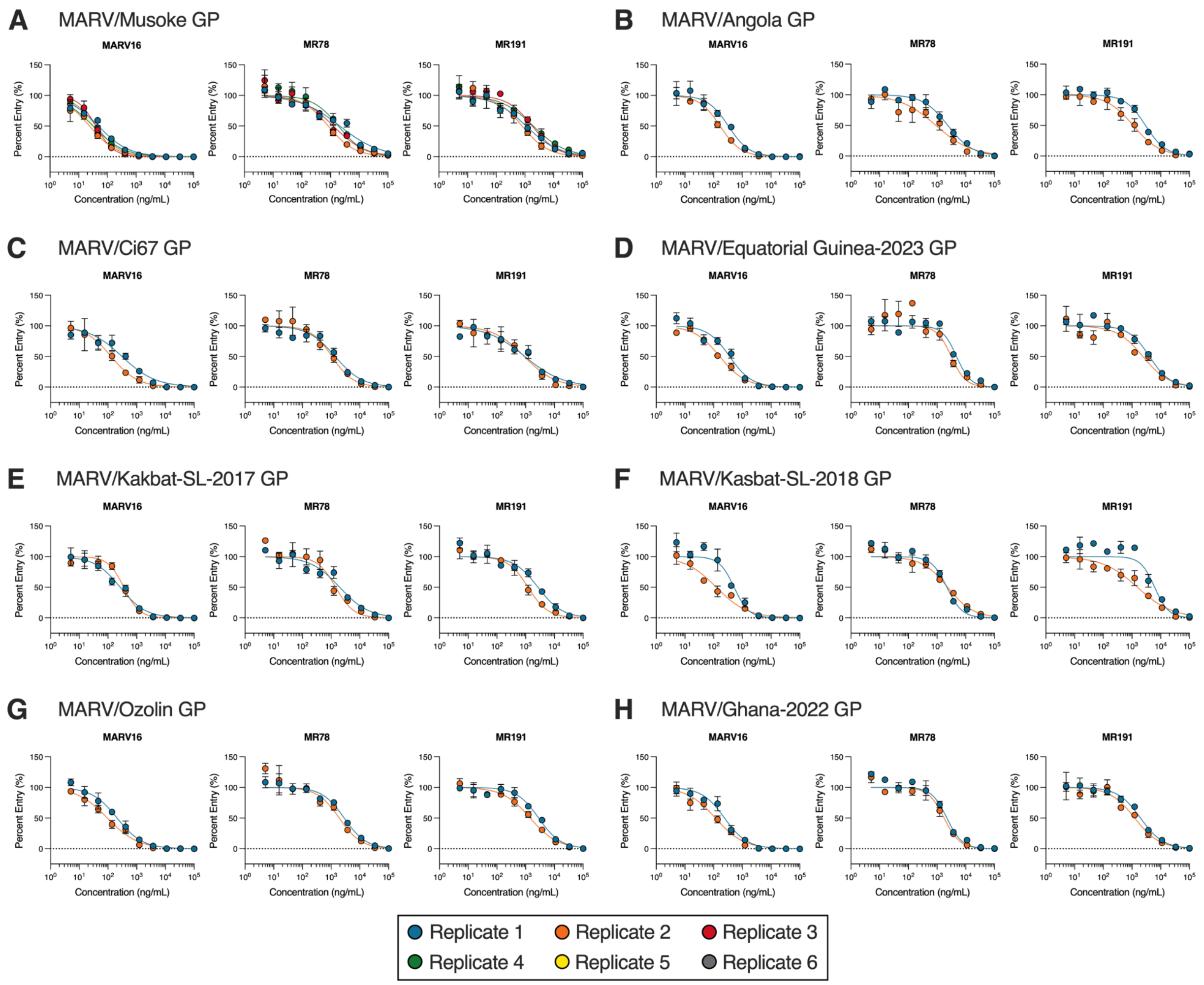


**Figure S4.** Neutralization dose-response curves for MARV16, MR78, and MR191 against VSV pseudotyped with the MARV/Musoke (A), MARV/Angola (B), MARV/Ci67 (C), MARV/Equatorial Guinea-2023 (D), MARV/Kakbat-SL-2017 (E), MARV/Kasbat-SL-2018 (F), MARV/Ozolin (G), or MARV/Ghana-2022 GP (H). Each of the two to six biological replicates used distinct batches of pseudoviruses and antibodies and data are shown as the mean ± standard error of technical triplicates.

bioRxiv preprint doi: https://doi.org/10.1101/2025.05.14.654121; this version posted May 18, 2025. The copyright holder for this preprint (which was not certified by peer review) is the author/funder, who has granted bioRxiv a license to display the preprint in perpetuity. It is made available under aCC-BY 4.0 International license.

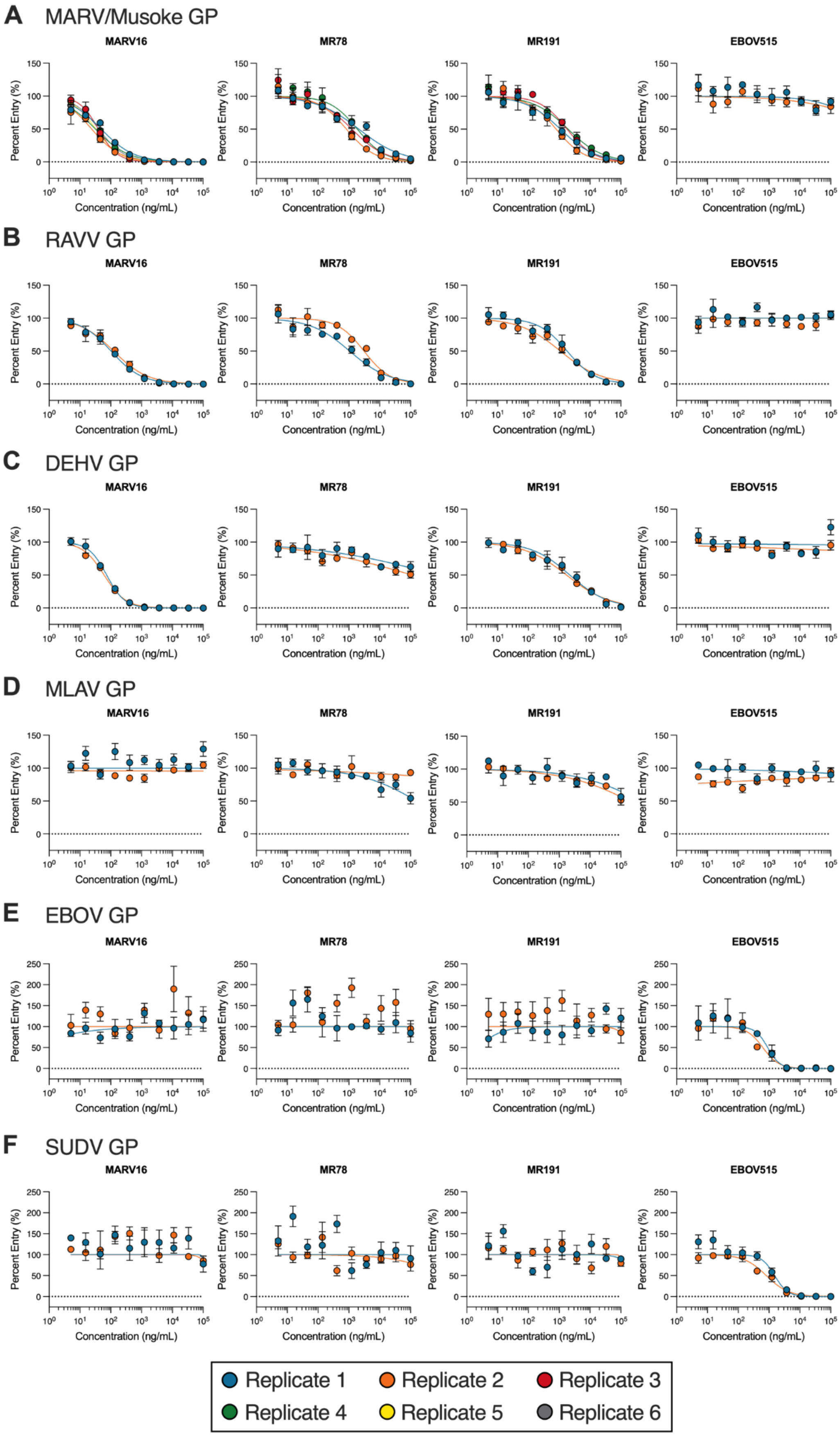

bioRxiv preprint doi: https://doi.org/10.1101/2025.05.14.654121; this version posted May 18, 2025. The copyright holder for this preprint (which was not certified by peer review) is the author/funder, who has granted bioRxiv a license to display the preprint in perpetuity. It is made available under aCC-BY 4.0 International license.

**Figure S5.** Neutralization dose-response curves for MARV16, MR78, MR191, and EBOV515 against VSV pseudotyped with the MARV/Musoke (A), RAVV (B), DEHV (C), MLAV (D), EBOV (E), or SUDV GP (F). Each of the two to six biological replicates used distinct batches of pseudoviruses and antibodies and data are shown as the mean ± standard error of technical triplicates.

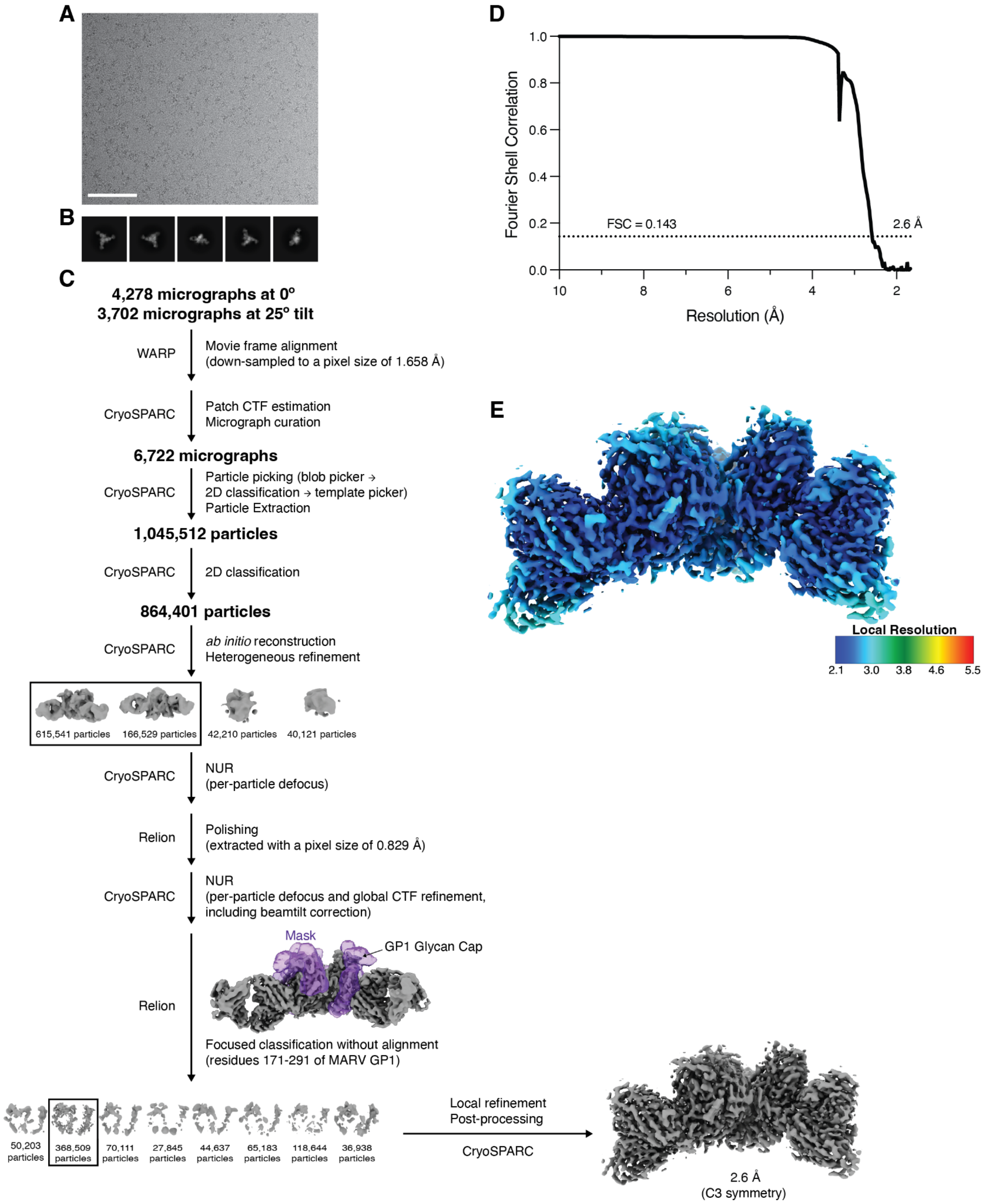

bioRxiv preprint doi: https://doi.org/10.1101/2025.05.14.654121; this version posted May 18, 2025. The copyright holder for this preprint (which was not certified by peer review) is the author/funder, who has granted bioRxiv a license to display the preprint in perpetuity. It is made available under aCC-BY 4.0 International license.

**Figure S6. A-B)** Representative cryo-EM micrograph (A) and 2D class averages (B) obtained for MARV GPΔMuc in complex with MARV16 Fabs. Scale bar: 100 nm. **C)** Cryo-EM data processing workflow. **D)** Gold-standard fourier shell correlation curves for the MARV GPΔMuc-MARV16 complex. **E)** Local resolution calculated with cryoSPARC for the locally refined MARV GPΔMuc-MARV16 complex (local filter map).

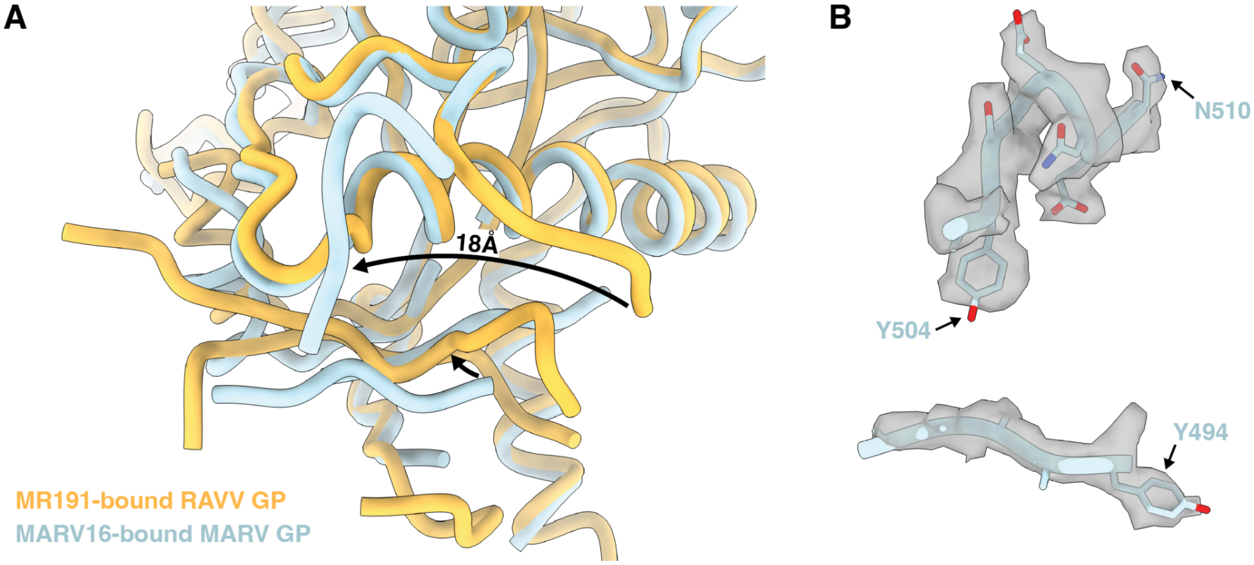


**Figure S7. A)** Superimposition of the MR191-bound RAVV GP (PBD: 6BP2; orange) and MARV16-bound MARV GP (blue) comparing the GP2 wing domain of the two models. The arrows indicate the position of the wing residues in each model. The MR191 and MARV16 Fabs are hidden for clarity. **B)** The MARV GP2 wing modeled into the cryo-EM density map (gray surface).

bioRxiv preprint doi: https://doi.org/10.1101/2025.05.14.654121; this version posted May 18, 2025. The copyright holder for this preprint (which was not certified by peer review) is the author/funder, who has granted bioRxiv a license to display the preprint in perpetuity. It is made available under aCC-BY 4.0 International license.

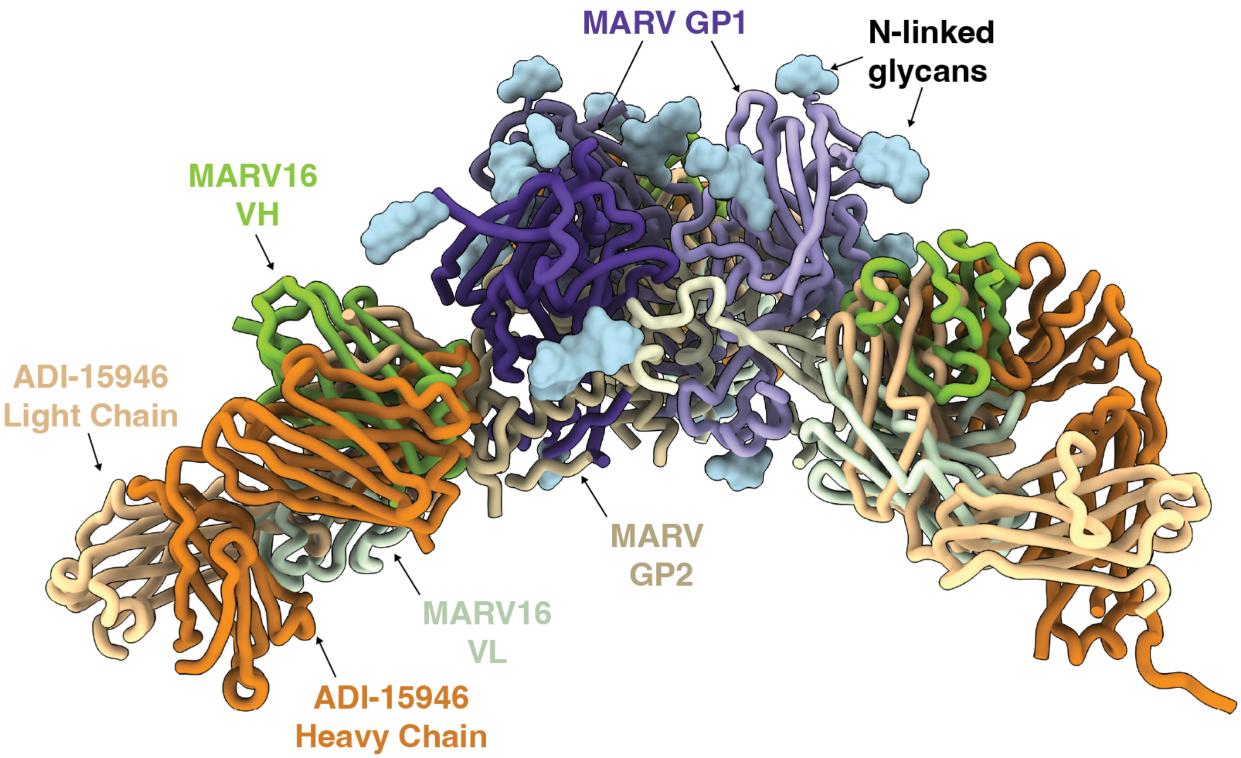


**Figure S8.** Comparison of the binding modes of MARV16 (green) and the pan-ebolavirus antibody, ADI-15946 (orange). The EBOV GP trimer from the EBOV GP-ADI-15946 complex structure(PDB: 6MAM) was superimposed with the MARV GP trimer from the MARV GPΔMuc-MARV16 structure to compare the ADI-15946 and MARV16 binding poses. MARV GP1 and GP2 are shown in different shades of purple and beige, respectively. N-linked glycans are rendered as cyan surfaces.

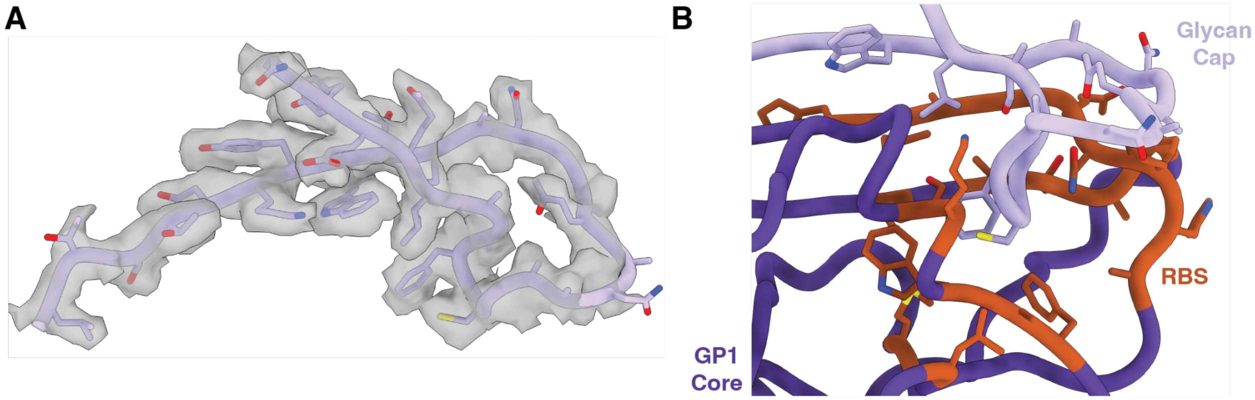


**Figure S9. A)** The MARV GP1 glycan cap (residues 191-219) modeled into the cryo-EM density map (gray surface). **B)** View of the putative RBS residues (shown in orange) that are shielded by the MARV GP1 glycan cap (light purple).

bioRxiv preprint doi: https://doi.org/10.1101/2025.05.14.654121; this version posted May 18, 2025. The copyright holder for this preprint (which was not certified by peer review) is the author/funder, who has granted bioRxiv a license to display the preprint in perpetuity. It is made available under aCC-BY 4.0 International license.

**Table S1.** Binding kinetics of the MARV16 Fab to immobilized MARV GPΔMuc. Values are presented as mean ± standard deviation obtained from two biological replicates using distinct batches of protein.

| $K_D$ (nM) | $k_{on}$ ($M^{-1}s^{-1}$) | $k_{off}$ ($s^{-1}$) |
|---|---|---|
| 1.35 ± 0.10 | 1.27 x $10^5$ | 1.70 x $10^{-4}$ |

**Table S2.** Cryo-EM data collection, refinement and validation statistics

| | MARV GPΔMuc-MARV16 (EMDB-xxxx) (PDB xxxx) |
|---|---|
| **Data collection and processing** | |
| Magnification | 105,000 |
| Voltage (kV) | 300 |
| Electron exposure (e–/Å$^2$) | 63 |
| Defocus range (μm) | -0.4 to -3.0 |
| Pixel size (Å) | 0.829 |
| Symmetry imposed | *C3* |
| Initial particle images (no.) | 1,045,512 |
| Final particle images (no.) | 368,509 |
| Map resolution (Å) | 2.6 |
| FSC threshold | 0.143 |
| | |
| **Refinement** | |
| Initial model used (PDB code) | 6BP2 |
| Model resolution (Å) | 2.6 |
| FSC threshold | 0.143 |
| Map sharpening *B* factor (Å$^2$) | -82.9 |
| Model composition | |
| Non-hydrogen atoms | 12,171 |
| Protein residues | 1,488 |
| Glycans | 39 |
| *B* factors (Å$^2$) | |
| Protein | 27.2 |
| Ligand | 68.7 |
| R.m.s. deviations | |
| Bond lengths (Å) | 0.003 |
| Bond angles (°) | 0.7 |

bioRxiv preprint doi: https://doi.org/10.1101/2025.05.14.654121; this version posted May 18, 2025. The copyright holder for this preprint (which was not certified by peer review) is the author/funder, who has granted bioRxiv a license to display the preprint in perpetuity. It is made available under aCC-BY 4.0 International license.

| | |
|---|---|
| Validation | |
| MolProbity score | 1.6 |
| Clashscore | 5.9 |
| Poor rotamers (%) | 2.1 |
| Ramachandran plot | |
| Favored (%) | 97.9 |
| Allowed (%) | 2.1 |
| Disallowed (%) | 0 |
